# Perception, Beliefs, and Causal Attribution of Autism Early Signs in Ecuadorian General Population

**DOI:** 10.3389/fpsyg.2022.915817

**Published:** 2022-06-23

**Authors:** Paulina Buffle, Edouard Gentaz, Giacomo Vivanti

**Affiliations:** ^1^Department of Psychology and Education, Université de Genève, Geneva, Switzerland; ^2^Swiss Center for Affective Sciences, University of Geneva, Geneva, Switzerland; ^3^AJ Drexel Autism Institute, Drexel University, Philadelphia, PA, United States

**Keywords:** autism, early signs, perceptions, causal attributions, culturally-sensitive practice, underrepresented groups, low-and middle-resource settings

## Abstract

The identification and diagnosis of children with autism currently rely on behavioral presentation and developmental history. Cultural norms and other socio-demographic factors can influence what is expected or non-expected behaviors in a developing child. Perceptions, beliefs, and causal attribution of early signs can influence families’ help-search behaviors. Lack of recognition of autism’s first manifestations can critically delay the age of diagnosis, the provision of informed guidance to families, and the implementation of adapted interventions during the critical period of early development. Furthermore, a lack of understanding of early signs as the manifestations of a developmental condition may increase stigma and non-conventional explanations. Still, cultural and socio-demographic factors are largely understudied, particularly in low-and middle-income settings. Based on the hypothesis that non-specialists such as family members and friends are one of the first sources of referral in Latin American contexts, we aimed to study the general population’s perceptions and the explanatory causes of autism’s early signs. One-hundred-and-eighty-three Ecuadorian adults responded to a questionnaire developed for this study, describing sixteen ASD-related behaviors. Results indicated that, with the exemption of language impairment and self-injurious behaviors, a substantial proportion of participants did not endorse many behaviors as “concerning and requiring professional attention.” Also, language impairment was the only behavior identified as related to a developmental disorder. Additionally, most participants attributed the majority of behaviors listed in the questionnaire to causes unrelated to ASD, such as child personality. We discuss the impact of those findings in clinical practice and on awareness programs.

## Introduction

Autism Spectrum Disorder (ASD) refers to a group of neurodevelopmental conditions characterized by difficulties in social communication, and unusually restricted or repetitive behavior and interests (American Psychiatric Association, 2013). Although support at any age is essential, younger children may benefit most from intervention due to early brain plasticity (Dawson et al., 2010; Vivanti et al., 2016). However, access to services may not be granted in all socioeconomic contexts (Durkin et al., 2015; Tekola et al., 2016). At least one in one hundred children in the world develops with a form of autism (Elsabbagh et al., 2012; Maenner et al., 2020), and around 6 million individuals live with this condition in Latin America (Paula et al., 2020).

Studies conducted in high-resource settings have reported several barriers to ASD-targeted diagnostic services, such as reduced availability of specialized services, lack of familiarity with tools, and perceptions of ASD as a not well-defined condition (Dosreis et al., 2006; Daniels and Mandell, 2013; Fenikilé et al., 2015). Additionally, socio-demographic and culturally determined factors, such as the knowledge the general population possesses about developmental milestones (Ratto et al., 2016), caregivers’ ability to recognize early emerging symptoms (Mandell and Novak, 2005), and misconceptions about signs, symptoms, and etiology (Rahbar et al., 2011) can play a role in trajectories toward identification. For instance, a study performed in the United States with mothers from a Latin-American origin reported significantly fewer developmental concerns and fewer ASD symptoms in children diagnosed with ASD in this group, compared to Anglo-American mothers, possibly due to lower ASD awareness, parental practices, or the presence of perceptions concealing the recognition of symptoms (Blacher et al., 2019). Another United States study with families of Mexican heritage reported that parents expect children to show respect for their elders by not speaking to them unless they are spoken to, suggesting that in this context, a lower level of communication might not be acknowledged as concerning (Bridges et al., 2012).

Similarly, a lack of response to parents’ directions might be interpreted as “willfulness” or be related to a child’s personality (Ratto et al., 2016). A reduced frequency of social initiations can be understood as a sign of politeness (“bien educado”), as has been reported in a study involving Mexican American children (Bridges et al., 2012). The same perception of a “good child” has been described in an Indian study, which has also documented that parents notice something atypical about their child six to ten months later than parents in the United States, suggesting that in this context, misleading causal attribution can impact on families’ help-seeking actions (Daley, 2004).

A low degree of concern and unconventional causal explanations may also impact the nature and quantity of information that caregivers provide to clinicians and professionals’ interpretation of this information (Mandell and Novak, 2005). Besides, these factors could also influence the choice of treatment. For example, a United States study suggested that non-conventional causal attributions result in parents seeking non-conventional treatments (Harrington et al., 2006). Another study has indicated that parents who believe food allergies are the explanatory cause of their child’s ASD symptoms may be more likely to use dietary modification and vitamins (Dardennes et al., 2011), and parents attributing those symptoms to vaccines are more likely to use detoxification therapy (Shyu et al., 2010). Hispanic parents in the United States have been reported to be less likely to endorse causal attributions that lead to help-seeking actions than non-Hispanic white American parents (Yeh et al., 2004). Similarly, non-white, publicly insured parents and parents with lower socioeconomic status have been reported to be less likely than others to interpret their child’s unusual behaviors as being caused by genetic factors, which possibly impact families’ help-seeking actions (Zuckerman et al., 2016).

These studies suggest that a better understanding of contextual factors, including perceptions of typical and atypical behaviors and culturally influenced explicative causes, is critical to inform awareness and mental health literacy programs supporting early diagnosis and intervention. However, most studies have been performed on causal explanations parents provide to their child’s autism (Hebert and Koulouglioti, 2010), and limited information is available on the perception of autism early behaviors among the general population, particularly in contexts different from those where the concepts and tools related to their identification have been developed.

### The Ecuadorian Context

Ecuador is an upper-middle-income country with a population under 18 years old, estimated at 6,298,788 in 2018 (National Institute of Statistics and Censuses of Ecuador, 2010). As preliminary evidence, a study aiming to estimate attendance of children with an autism diagnosis at schools in Quito found a proportion of 0.11% among 453 pupils, ages 5 to 15, in 161 regular schools (Dekkers et al., 2015). Health authorities estimate a prevalence of 0.28% (0.18%–0.41%) in children aged five years old or less, and 1266 people diagnosed with ASD were reported in official registers (Ministry of Public Health, 2017, p. p. 11). The reason why estimates of ASD prevalence in Ecuador are remarkably lower than those reported in Western Countries (Elsabbagh et al., 2012; Zeidan et al., 2022) remains unclear.

As has been the case in many countries, parent associations in Ecuador have contributed to increasing the understanding of the needs of the autism spectrum community through public conferences and participation in the development of social policies (Buffle, 2020). In 2012, the Ministry of Public Health recognized autism as a disability (Ministry of Public Health, 2012), a status that grants specific rights to individuals with a diagnosis. The same year, a law on disabilities was adopted, aiming to ensure the prevention, detection, treatment, and rehabilitation and to assure that the rights of people with disabilities are guaranteed (National Assembly of the Republic of Ecuador, 2012). An agreement intending to ensure access to special education was enacted in 2013 (Ministry of Education, 2013).

More recent efforts include the publication of an official guide that provides information on identification and treatment in clinical practices (Ministry of Public Health, 2017) and the definition of a National Agenda 2017–2021 aiming to support the autonomy and productivity of people with disabilities (National Council for Equality in Disabilities, 2017). Universities offer postgraduate education in pediatrics; however, the specific pedo-psychiatric and neuro-pediatric fields rely on interns trained in other countries who return to work in Ecuador. In the pediatric sector, professionals may face many challenges in ASD case-identification and case-management, such as access to training and available resources to refer families (Buffle et al., 2022). The public health system has two pediatric hospitals in two main cities, and several community centers around the county and units providing specific ASD services of identification and treatment are currently being developed.

Understanding the general population’s perceptions and interpretation of symptoms in this context is essential to inform both educational programs for specialists and local awareness programs. This understanding is particularly relevant in low-to-middle income contexts, where toddlers may not be seen systematically for well-being child visits, and child-health programs may focus predominantly on life-threatening issues with less attention to developmental disabilities (Elsabbagh et al., 2012). Moreover, in the Latin American context, non-specialists in health or education, such as family members and friends, are reported to be one of the first sources of referral for children with autistic symptoms (Talero-Gutiérrez et al., 2012), and the Internet is reported as one of the primary sources of information for families of individuals with ASD (Paula et al., 2020).

To the best of our knowledge, perceptions and causal attribution of ASD symptoms in young children have not been documented in Latin American countries. In the current study, we used a questionnaire with items about social communication, play, and disruptive behaviors described in the literature presented to a group of adults from the general population. Based on the hypothesis that in Ecuador, as in many other countries, awareness of ASD symptoms is limited, we expected that a minority of ASD characteristic behaviors would be identified as “concerning and requiring professional attention” and that a minority of behaviors would be understood as the manifestation of a developmental condition. We also examined the types of explanations provided for each behavior. Finally, we examined whether demographic factors influenced participants’ endorsement of ASD-related behaviors as “concerning” and as manifestations of a neurodevelopmental disorder.

## Materials and Methods

### Instrument

The description of behaviors presented in the questionnaire was inspired by 18 behaviors related to social communication and play described in the literature (Barbaro and Dissanayake, 2009; Robins et al., 2009; Barbaro et al., 2011) and available in Spanish (Albores-Gallo et al., 2012). In order to present a wide range of autism-related behaviors, the questionnaire also included one item related to expressive language delay, reported as one of the first signs alerting parents in different cultures (De Giacomo and Fombonne, 1998; Daley, 2004). Two questions about challenging behaviors that can be present in some young children developing with ASD (Matson et al., 2010, 2011) were also included.

A group of ten local advisors (five parents of toddlers and school-age children, two kindergarten teachers, two pediatricians, and one anthropologist) was required to verify each of the 21 items in terms of understandability and adaptability to an Ecuadorian context. Five advisors perceived five items (four related to social and communication behaviors and one related to make-believe play) as “abstract” and “difficult to understand for adults who are not familiar with children” or “difficult to understand without actually *seeing* the behavior,” These behaviors were excluded from the questionnaire.

The sixteen behaviors described in the questionnaire included: three joint attention behaviors, nine social communication behaviors (including expressive, receptive language), two idiosyncratic responses to sensory stimuli and unusual motor patterns, one behavior related to play and two challenging behaviors.

Considering the variability of the onset of symptoms during the first years of life (American Psychiatric Association, 2013) and aiming to assure a more precise mental representation of the items described, the questionnaire specified that the first eight items concerned children between 18 and 24 months; the remaining items concerned children aged between 24 and 36 months (Barbaro and Dissanayake, 2009; Robins et al., 2009; Boyd et al., 2010; Barbaro et al., 2011).

As an essential aim in the first section of the questionnaire was to capture the intensity of concerns elicited by the depicted behaviors, participants were required to select one of three options: (a) the behavior depicted corresponds to what is “expected at that age,” (b) the behavior is “peculiar, but it does not need for professional attention,” or (c) the behavior is “concerning and requiring professional attention.” The second section of the questionnaire aimed to collect participants’ causal attribution for each behavior through an open-ended question.

### Participants

One hundred eighty-three adults (107 female) between 18 and 79 years old (M_age_ = 36.63, age-range = 18–79) from different socioeconomic statuses, professional and educational backgrounds, reporting to be born or raised in Ecuador, were enrolled through face to face recruitment (Table 1).

**TABLE 1:** Sample characteristics (*n* = 183).

Characteristics	n
**Gender**	
Male	76
Female	107
**Age (in years)**	
18–30	87
31–79	96
**Having children**	
No child	40
One child	143
**Education level**	
Primary school	46
Secondary school	36
Preparatory or Professional School	35
Basic or advanced university degree	66
Primary work activity*	
Managers	10
Professionals	25
Technicians and associate professionals	20
Clerical support workers	19
Service and sale workers	19
Skilled agricultural, forestry, and fishery Workers	5
Craft and related trades workers	17
Plant and machine operators and assemblers.	10
Elementary occupations	28
Not employed	30

*Professional background of participants was obtained and classified following the International Standard Classification of Occupations of the International Labor Office, adapted to Ecuador (National Institute of Statistics and Censuses of Ecuador, 2010).

In order to increase variability in the population, a list of popular markets attended by lower-middle to low-income classes was downloaded from a government agency,^1^ and a list of supermarkets attended by the upper-middle-income class was established. Five sites of each category were selected in 4 different towns, including the capital city, Quito, and permission to display information and proceed to interviews was obtained.

Participants were individually invited to participate and received information on the aim and procedure of the study. The information provided avoided using terms related to autism to exclude bias. Approximately 5% of individuals who approached the researchers for information declined the invitation to participate after receiving the information. Participants who accepted to participate provided written consent and were interviewed in Spanish by the author or by a graduate student in anthropology, both fluent in the language and familiar with Ecuadorian culture.

The 16-items were orally presented to avoid situations where participants could not read. Each autistic-related behavior was described to the participants, who were given enough time to produce a mental image of the situation described. Then, the two questions were read to the same participant. The researcher wrote down the option chosen for the first question (degree of concern) and the response to the open-ended question (causal attribution of the behaviors). The question related to the degree of concern and the question related to the explanatory cause were identical for each of the sixteen behaviors; participants responded in approximatively 30–35 min to the entire questionnaire. Participants also responded to a short demographic questionnaire. The research ethics committee of the first author provided approval for the study.

### Data Analysis

The responses to 16 open-ended questions were transcribed verbatim and classified using content analysis, a valuable method to identify emerging categories or themes from participants’ responses (Elo and Kyngäs, 2008; Kvale, 2008). The principal researcher, fluent in Spanish, read transcripts several times, aiming to identify regional and colloquial expressions. Local expressions were clarified by a local assistant (a nurse student). Each response was then introduced into an Excel sheet with responses in one column and the categories in the next column, and a code was assigned for each pattern identified. Constructed categories corresponded to ideas or expressions common among the participants’ responses.

In a second phase, the first researcher and a Spanish-speaker postgraduate student in anthropology independently verified that each response was assigned to at least one of the different constructed categories. Aiming to ensure reliability, a medical resident fluent in Spanish and blind to hypotheses independently reviewed by double-checking the constructed categories. Conflict situations (8% of responses) were resolved after discussion.

Eight categories were defined during the process of coding. (1) Personality Attitudes and Preferences; (2) Developmental Problems; (3) Physical or Sensory Issues; (4) Psychological Problems; (5) Rearing/Parenting; (6) Unspecified Causes; (7) Immaturity; (8) Does Not Know. Finally, frequencies of responses were calculated for all closed-ended and open-ended questions for each of the 16 behaviors.

## Results

### Perception of Autism-Related Behaviors

As shown in Table 2, the two behaviors most frequently endorsed as “concerning and requiring professional attention” were Doesn’t Speak – Only Produce Sounds, and *Hits his Head with Hands or Objects*. Key social communication behaviors, such as *Doesn’t Respond to Name*, *Doesn’t have Interest in Other Children*, *Doesn’t Understanding Emotions*, and *Doesn’t Understand Instructions*, also received a high endorsement as “concerning” behaviors. The behavior that attracted less concern was *Play Concentrates on Specific Toy Parts.*

**TABLE 2:** Perception of the degree of concern by 183 adults in % in function of 16 behaviors.

Behaviors	Concerning		Peculiar		Expected		DN	
	n	%	n	%	n	%	n	%
1. Doesn’t respond to pointing	62	34	78	43	42	23	1	1
2. Doesn’t point to desired objects	62	34	83	45	37	20	1	1
3. Doesn’t point at interesting objects	60	33	86	47	38	20	0	0
4. Doesn’t respond to name	79	43	68	37	33	18	3	2
5. Avoids eye contact	48	26	47	86	48	26	1	1
6. Play concentrates on specific toy parts	33	18	60	33	88	48	2	1
7. Doesn’t handle interesting objects to adults	48	26	85	46	49	27	1	1
8. Doesn’t imitate adults	37	20	102	56	44	24	0	0
9. Doesn’t speak – only produce sounds	114	62	47	26	22	12	0	0
10. Doesn’t have interest in other children	77	42	72	39	34	19	0	0
11. Doesn’t understand instructions	73	40	69	38	40	22	1	1
12. Doesn’t understand emotions	76	42	63	34	41	22	3	2
13. Produce unusual finger movements	52	28	63	34	67	37	1	1
14. Covers ears to common sounds	37	20	68	37	76	42	2	1
15. Hits his head with hands or objects	113	62	53	29	17	9	0	0
16. Angers abruptly if environment changes	26	48	62	34	71	39	2	1

The three joint attention behaviors presented, Doesn’t Respond to Pointing, Doesn’t Point at Desired Objects, and Doesn’t Point at Interesting Objects, were considered by most respondents as peculiar but not sufficiently alarming to search for professional advice. Two other behaviors related to social-communicative difficulties that were also considered peculiar are Doesn’t Imitate, Avoids Eye Contact, and Doesn’t Show Interesting Objects to Adults. Finally, the behaviors mostly considered “expected” were Play Concentrates on Specific Toy Parts, Covers Ears to Common Sounds, Angers Abruptly if Environment Changes, and Produce Unusual Finger Movements.

### Causal Attribution of Autism-Related Behaviors

The explanatory cause most commonly cited (ten out of sixteen behaviors) was Personality, Attitude, or Preferences (Table 3). Some examples include “Es caprichoso” [He is spoiled] or “Es de temperamento” [He is temperamental]. The category Developmental Problems was the leading explanatory cause for only one of the sixteen behaviors, namely Doesn’t Speak – Only Produce Sounds. In this case, the specificity of the participant’s responses ranged from “Está atrazado” [His development is delayed] to precise answers such as “Tiene un trastorno del desarrollo” [He has a developmental disorder]. To a lesser extent, Hits his Head with Hands or Objects, Doesn’t Respond to Pointing, and Doesn’t Understand Emotions were understood as a sign of special needs situation: “Es una situación de necesidades especiales.”

**TABLE 3:** Causal attributions repartition in eight categories by 183 adults in % in function of 16 behaviors.

Behaviors	1		2		3		4		5		6		7		8	
	n	%	n	%	n	%	n	%	n	%	n	%	n	%	n	%
1. Doesn’t respond to pointing	40	22	34	19	35	19	0	0	8	4	5	3	18	10	43	23
2. Doesn’t point to desired objects	22	12	22	12	15	8	0	0	13	7	17	9	29	16	65	36
3. Doesn’t point at interesting objects	35	19	24	13	9	5	1	1	5	3	7	4	24	13	78	43
4. Doesn’t respond to name	19	10	13	7	81	45	0	0	4	2	2	1	19	10	45	25
5. Avoids eye contact	54	30	20	11	13	7	4	2	2	1	2	1	19	10	69	38
6. Play concentrates on specific toy parts	47	26	13	7	9	5	0	0	2	1	9	5	17	9	86	47
7. Doesn’t handle interesting objects to adults	49	27	24	13	2	1	0	0	6	3	8	4	21	11	73	40
8. Doesn’t imitate adults	48	26	21	11	6	3	3	2	4	2	6	3	33	18	62	34
9. Doesn’t speak – only produce sounds	4	2	84	46	14	8	2	1	11	6	9	5	30	16	29	16
10. Doesn’t have interest in other children	73	40	16	9	1	1	8	4	4	2	6	3	31	17	44	24
11. Doesn’t understand instructions	43	23	21	11	31	17	2	1	6	3	9	5	23	13	48	26
12. Doesn’t understand emotions	17	9	30	16	15	8	4	2	5	3	10	5	37	20	65	36
13. Produce unusual finger movements	21	11	27	15	27	15	2	1	1	1	15	8	27	15	63	34
14. Covers ears to common sounds	54	30	6	3	38	21	2	1	1	1	12	7	7	4	63	34
15. Hits his head with hands or objects	42	23	36	20	14	8	18	10	0	0	0	0	32	17	41	22
16. Angers abruptly if environment changes	64	35	6	3	2	1	9	5	8	4	5	3	17	9	72	39

*Eight constructed categories are defined as follows: 1: Personality Attitudes and Preferences; 2: Developmental Problems; 3: Physical or Sensory Issues; 4: Psychological Problems; 5: Rearing/Parenting; 6: Unspecified Causes; 7: Immaturity; 8: Does Not Know.*

Physical or Sensory Issues explanations such as, “No oye bien” [He doesn’t hear properly], were mainly associated with Doesn’t Respond to Name and Cover Ears in Response to Common Sounds. Scarce explanations were provided for the category Psychological Problems; examples included “Está traumado” [He is traumatized] or “Está estresado” [He is stressed] and were mainly associated with Hits his Head with Hands or Objects. Scarce explanations were also provided in the category Parenting; for example, “Sus padres no le han enseñado” [His parents haven’t taught him] was provided to the behavior Doesn’t Point to Desired Objects. The category Unspecified Reasons assembled responses that acknowledge a behavior as unusual but did not provide an explanatory cause, such as “Algo le pasa” [Something is happening to him], “Algo hay” [There is something]. Supranatural explanatory causes were provided by less than 1% of participants and were also included in this category. Explanatory causes related to the category Immaturity, such as “Todavía es chiquito” [He is still young], were provided by a small proportion of participants for all the behavior presented. Finally, an important rate of responses was associated to the category I don’t know and was provided to most of the behaviors presented.

### Difference of Proportions

We also aimed to study if the level of education, age, and parental status influenced the identification of behaviors as “concerning and requiring professional attention” and the explanatory causes related to a developmental condition.

In order to explore the effect of these factors, participants were classified by age (group 1 = 18–30 years old; group 2 = 31–79 years old), parenthood status (group 1 = parents; group 2 = no parents), and level of education (group 1 = primary and secondary education; group 2 = university and post-degree). The data was coded and entered into Statistica (Tibco) to analyze the differences between proportions testing the following hypotheses *H*_0_:*P*_*O*_ = *P_Y_*.

*H*_1_: PO > *P_Y_* with a level of significance of 5%. Z-value equals the observed difference between proportions (p1-p2) divided by the standard error:


$$\text{z}=\frac{p_{1}-p_{2}}{\sqrt{p\,q\,\left(\frac{1}{n_{1}}+\frac{1}{n_{2}}\right)}}=\frac{p_{1}-p_{2}}{S\,E}$$

Classification under age indicated a significant impact on the perception as *“concerning*” among older participants on the following behaviors: Avoids Eye Contact, Doesn’t Handle Interesting Objects to Adults, Doesn’t Have Interest in Other Children, Doesn’t Understand Instructions, Doesn’t Understand Emotions. Parental status had a significant impact on the perception as “concernin” among older participants only on Avoids Eye Contact. The level of education was not found to have an effect on the perception of any of the behaviors as “concerning” (Table 4).

**TABLE 4:** Differences of proportions of participants that identified the 16 behaviors as “concerning” as a function of age, parenthood, and level of education.

	Age					Have children				Level of education					
Behaviors	18–30		31–79			Yes		No			Low		High		
	*n* = 87		*n* = 96			*n* = 87		*n* = 96			*n* = 82		*n* = 101		
	n	%	n	%	p	n	%	n	%	p	n	%	n	%	p
1. Doesn’t respond to pointing	17	20	20	21	0.413	19	18	18	23	0.797	18	22	19	19	0.701
2. Doesn’t point to desired objects	12	14	21	22	0.078	21	20	12	15	0.211	15	18	18	18	0.533
3. Doesn’t point at interesting objects	19	22	29	30	0.099	30	29	18	23	0.202	23	28	25	25	0.693
4. Doesn’t respond to name	29	33	31	32	0.560	36	34	24	31	0.308	22	27	38	38	0.061
5. Avoids eye contact	39	45	75	78	**0.000**	73	70	41	53	**0.010**	55	67	59	59	0.885
6. Play concentrates on specific toy parts	35	40	41	43	0.367	43	41	33	42	0.573	35	43	41	41	0.612
7. Doesn’t handle interesting objects to adults	26	30	51	53	**0.001**	48	46	29	37	0.124	35	43	42	42	0.560
8. Doesn’t imitate adults	31	36	42	44	0.131	42	40	31	40	0.486	33	40	40	40	0.535
9. Doesn’t speak – Only produce sounds	25	29	37	39	0.081	38	36	24	31	0.222	31	38	31	31	0.844
10. Doesn’t have interest in other children	18	21	34	35	**0.014**	33	31	19	24	0.147	22	27	30	30	0.334
11. Doesn’t understand instructions	14	16	34	35	**0.001**	31	30	17	22	0.120	24	29	24	24	0.800
12. Doesn’t understand emotions	31	36	48	50	**0.025**	48	46	31	40	0.210	34	41	45	45	0.337
13. Produce unusual finger movements	16	18	21	22	0.278	19	18	18	23	0.797	16	20	21	21	0.415
14. Covers ears to common sounds	21	24	27	28	0.271	29	28	19	24	0.310	23	28	25	25	0.693
15. Hits his head with hands or objects	32	37	30	31	0.785	38	36	24	31	0.222	24	29	38	38	0.167
16. Angers abruptly if environment changes	49	56	64	67	0.075	69	66	44	56	0.100	53	65	60	60	0.765

*p < 0.05 shown in bold.*

Results also indicated that the age of the participants had a significant influence on the recognition of five behaviors as a *sign of a developmental disorder*: Avoids Eye Contact and Doesn’t Handle Interesting Objects to Adults, with older participants more often endorsing these behaviors as “concerning.” Participants with children provided a *developmental explanatory cause* to Covers Ears to Common Sounds and Doesn’t Handle Interesting Objects more often. Participants with a higher level of education provided a *developmental explanatory cause to* Produce Unusual Finger Movements more often. The level of education seemed to have no impact on understanding any of the behaviors as a symptom of a *developmental disorder* (Table 5). The results of this exploratory analysis should be interpreted with caution, considering the sample size.

**TABLE 5:** Differences of proportions of participants identifying 16 behaviors as a symptom of a developmental disorder, as a function of age, parenthood, and level of education.

	Age					Have children					Level of education				
Behaviors	18–30 ans		31–79			Yes		No			Low		High		
	*n* = 87		*n* = 96			*n* = 87		*n* = 96			*n* = 82		*n* = 101		
	n	%	n	%	p	n	%	n	%	p	n	%	n	%	p
1. Doesn’t respond to pointing	4	5	2	2	0.829	4	4	2	3	0.320	4	5	2	2	0.863
2. Doesn’t point to desired objects	6	7	7	7	0.460	9	9	4	5	0.185	6	7	7	7	0.540
3. Doesn’t point at interesting objects	9	10	15	16	0.145	14	13	10	13	0.459	10	12	14	14	0.370
4. Doesn’t respond to name	9	10	15	16	0.145	16	15	8	10	0.162	10	12	14	14	0.370
5. Avoids eye contact	29	33	55	57	**0.001**	53	50	31	40	0.075	33	40	51	50	0.083
6. Play concentrates on specific toy parts	14	16	16	17	0.460	17	16	13	17	0.534	11	13	19	19	0.163
7. Doesn’t handle interesting objects to adults	4	5	12	13	**0.029**	13	12	3	4	**0.022**	8	10	8	8	0.669
8. Doesn’t Imitate Adults	9	10	12	13	0.323	12	11	9	12	0.509	12	15	9	9	0.886
9. Doesn’t speak – only produce sounds	14	16	20	21	0.206	19	18	15	19	0.577	17	21	17	17	0.750
10. Doesn’t have interest in other children	14	16	13	14	0.688	13	12	14	18	0.853	13	16	14	14	0.647
11. Doesn’t understand instructions	10	11	10	10	0.591	11	10	9	12	0.590	7	9	13	13	0.175
12. Doesn’t understand emotions	7	8	6	6	0.681	7	7	6	8	0.996	5	6	8	8	0.317
13. Produce unusual finger movements	10	11	11	11	0.504	10	10	11	14	0.832	3	4	18	18	0.**001**
14. Covers ears to common sounds	1	1	5	5	0.062	6	6	0	0	**0.016**	5	6	1	1	0.973
15. Hits his head with hands or objects	11	13	11	11	0.599	15	14	7	9	0.137	11	13	11	11	0.699
16. Angers abruptly if environment changes	16	18	20	21	0.337	25	24	11	14	0.051	17	21	19	19	0.627

*p < 0.05 shown in bold.*

## Discussion

This study was designed to identify the perceptions of ASD early markers and the type of causal explanations the general adult population attributes to those behaviors. Several early ASD symptoms described in our questionnaire were perceived as “peculiar” although not “concerning” by at least one-third of the participants. These findings suggest that some common early signs could be overlooked by caregivers and adults surrounding a child. They are consistent with previous findings from Latin American communities in the United States reporting that mothers of children with ASD tend to normalize their child’s early behavior compared to non-Latin American mothers, which can negatively influence help-seeking actions (Zuckerman et al., 2014).

Our results also denote that participants did not perceive most of the behaviors presented in the questionnaire as potential manifestations of a developmental disorder, which is also consistent with previous studies on causal attribution of developmental difficulties (Zuckerman et al., 2016). For instance, in our sample, most of the participants could not provide a causal attribution to common autism-related behaviors, such as *Avoids Eye Contact* and *Doesn’t Handle Interesting Objects to Adults*. When an explicatory cause was provided, it was mainly related to the child’s personality. Interestingly, these two core symptoms have been reported among the first parental concerns in other low-and-middle-resources settings (Daley, 2004); however, in our sample, they were mainly endorsed as non-concerning. Those findings suggest that subtle behaviors associated with autism might pass unnoticed to adults in Ecuador and that pediatric providers should not assume that parents can spontaneously report them as a concern.

Similarly, the three joint attention behaviors (*Doesn’t Respond to Pointing, Doesn’t Point at Desired Objects*, and *Doesn’t Point at Interesting Objects*) were not considered “concerning,” and, as in the former example, most of the responders could not provide an explanatory cause or attributed it to a child’s personality. Those findings have a critical implication for clinical practice considering that they are commonly present in children who will receive a diagnosis. Furthermore, attribution of autistic symptoms to a child’s personality as “timidity,” “independence,” or “strong character” may have at least two potential impacts. Firstly, if caregivers and family friends underscore those behaviors parental help-seeking actions could be delayed. Secondly, rearing style can be affected, for example, if parents understand those behaviors as their child not wanting to cooperate or is voluntarily ignoring them. The same conclusion could be drawn for the items *Doesn’t Have Interest in Other Children* and *Doesn’t Understand Instructions*, which are considered “concerning” but mainly explained as the manifestation of a child’s personality.

*Lack of response to name* was identified as “concerning” by many participants but was primarily related to a hearing impairment, a type of explanation that may be pertinent in some cases and requires a differential diagnosis; however, in ASD cases, it has been associated with an increase in the age of diagnosis in the United States (Mandell et al., 2005). This finding points to the importance of continuous education for all pediatric health allies and in all levels of attention as children displaying atypical behaviors may be referred in the first instance to sensory examinations. Equally acknowledged as “concerning,” *self-injurious behaviors* were not identified as signs of a developmental disorder but rather as a personality problem. These perceptions could lead caregivers not to seek professional advice even though emerging self-injurious behavior can be associated with unfavorable outcomes and can be addressed through intervention.

The item *Play Concentrates on Specific Toy Parts*, which in the United States has been associated with a decrease in the age of diagnosis (Mandell et al., 2005), was considered by our sample as the least concerning behavior suggesting that interpretation of play-related behaviors may be under the contextual influence. The item *Covers Ears to Common Sounds*, *Angers Abruptly if Environment Changes*, was mainly perceived as expected and principally explained by personality. In the case of *Unusual Finger Movements*, participants provided several reasons unrelated to a developmental disorder, such as boredom, and it was mainly expected in typical development. Interestingly, these three behaviors are not related to social communicative abilities but rather to repetitive behaviors and sensory issues. It is difficult, at this stage, to know if the Ecuadorian population tends to identify non-social communicative ASD markers as less problematic and if those results reflect more tolerance of diversity in repetitive play and sensory issues. Further research is also needed to understand if identifying behaviors as problematic can help children and families in Ecuador access services or whether there is a risk of pathologizing certain behaviors in children that could otherwise be accepted and adjusted within their communities. A higher level of concern elicited by the social communicative markers is also consistent with the findings of a study performed in a group of Latin American countries, where more than half of the parents reported communication and social interaction deficits as the main challenges for their child with ASD (Paula et al., 2020).

*Expressive language delay*, as anticipated, was mainly endorsed as “concerning” and attributed to a developmental disorder suggesting that this difficulty could prompt families to seek professional consultation. However, because many children with ASD do not have a language delay, the presence of language could be a factor of late referral.

A very modest rate of supranatural explanations was found in our sample and was classified as unspecified causes. This finding is congruent with recent research. For example, a Canadian study found that the most common causal attribution provided by Latin American parents of children diagnosed with ASD was not related to religious explanations (Millau et al., 2018), and a study in the Philippines indicated that parents tend to disagree with previous myths about the etiology of ASD, such as parental sins and curses (Quilendrino et al., 2015).

Preliminary analyses did not suggest a clear association of perception of behaviors as “concerning” or as a sign of a developmental disorder and socio-demographic factors. Although the sample size limits these results, they suggest that awareness of ASD symptoms may be needed across all segments of the population in Ecuador, without distinction of age, parental status, or education. Community awareness remains an essential concern for families in Latin America despite significant recent efforts deployed (Paula et al., 2020). Awareness of the condition and its early manifestations is critical for the process of detection; it might also influence attitudes toward individuals with ASD and contribute to avoiding stigma.

These results suggest that parent education programs remain necessary, even if retrospective studies have shown that parents recognize signs of autism far earlier than it is diagnosed, particularly when they have an older child with autism (Ozonoff et al., 2009). Education aiming to inform parents about what to expect from their developing children during their early months and years could also help them identify when a child fails to reach certain milestones. Parents’ knowledge and understanding of early markers as the signs of a developmental condition can also influence their trust in diagnostic and willingness to comply with adapted early interventions (Mire et al., 2017). Finally, our results can inform professionals in pediatric settings about behaviors that caregivers may not consider “concerning and requiring professional attention,” which can impact the type of information parents transmit spontaneously during a consultation.

Generalizing those findings to other Latin American cultural contexts requires more extensive and more diversified participation. Although Latin American individuals share a language and many cultural references, they belong to a range of cultural sub-groups resulting from a complex interplay of self-perceived identities, socioeconomic factors, and level of education.

This study also presents methodological limitations related to some items, for example, *Produce Unusual Finger Movement*, which might be difficult to understand or visualize by non-specialists. This is an important point for the cross-cultural validity of parent-addressed checklists. As those instruments are essentially based on the description of behaviors, the understandability of such descriptions in various populations may need further study. Also, the questionnaire describes behaviors that could be present in other conditions, such as sensory impairments, receptive language disorder, or epilepsy (Wadhera, 2021), which could impact participants’ responses. Finally, it is important to notice that the behaviors presented in our questionnaire correspond to symptoms that have been defined as a “male stereotype” (Frazier et al., 2014).

Furthermore, all questions were related to behaviors that can be displayed in “un niño,” a masculine noun used as a generic for “a child” in Spanish, that could produce a gender bias as participants visualize these symptoms only in young boys. Further studies will need to be conducted to observe if the description of these behaviors elicits different degrees of concern and other causal explanations if applied to young girls. Further studies are also needed to determine if the concern elicited by certain behaviors, the type of explanation provided, or the presence of certain demographic traits could predict families’ help-seeking actions.

## Data Availability Statement

The raw data supporting the conclusions of this article will be made available by the authors, without undue reservation.

## Ethics Statement

The studies involving human participants were reviewed and approved by the Ethical Committee of the Faculty of Psychology and Education at Geneva University approved this study. The patients/participants provided their written informed consent to participate in this study.

## Author Contributions

PB and EG designed the study and analyzed the data. PB acquired the data. PB and GV wrote the article’s first draft. All authors have approved the final manuscript.

## Conflict of Interest

The authors declare that the research was conducted in the absence of any commercial or financial relationships that could be construed as a potential conflict of interest.

## Publisher’s Note

All claims expressed in this article are solely those of the authors and do not necessarily represent those of their affiliated organizations, or those of the publisher, the editors and the reviewers. Any product that may be evaluated in this article, or claim that may be made by its manufacturer, is not guaranteed or endorsed by the publisher.

## Footnotes

1
http://www.comercio.quito.gob.ec
