# Erratum: Perception, beliefs, and causal attribution of autism early signs in Ecuadorian general population

**DOI:** 10.3389/fpsyg.2023.1182237

**Published:** 2023-03-17

**Authors:** Frontiers Production Office

**Affiliations:** Frontiers Media SA, Lausanne, Switzerland

**Keywords:** autism, early signs, perceptions, causal attributions, culturally-sensitive practice, underrepresented groups, low-and middle-resource settings

An omission to the funding section of the original article was made in error. The following sentence has been added: “Open access funding was provided by the University of Geneva.”

The original article has been updated.

